# Supplementary material for: The nuclear receptor ERβ engages AGO2 in regulation of gene transcription, RNA splicing and RISC loading
Source: Genome Biol. 2017 Oct 6;18:189. doi: 10.1186/s13059-017-1321-0 (PMC5634881; doi:10.1186/s13059-017-1321-0)
Supplement: Supplementary file 6 — Supplementary figures with legends. (DOCX 3897 kb) [file 13059_2017_1321_MOESM6_ESM.docx]

**Additional file 6**

**The nuclear receptor ERbeta engages AGO2 in regulation of gene transcription, RNA splicing and RISC loading to exert its oncosuppressor activities in breast cancer cells**

*Tarallo et Al.*

**Figures S1-S7**

**Figure legends**

**Figure S1**

**
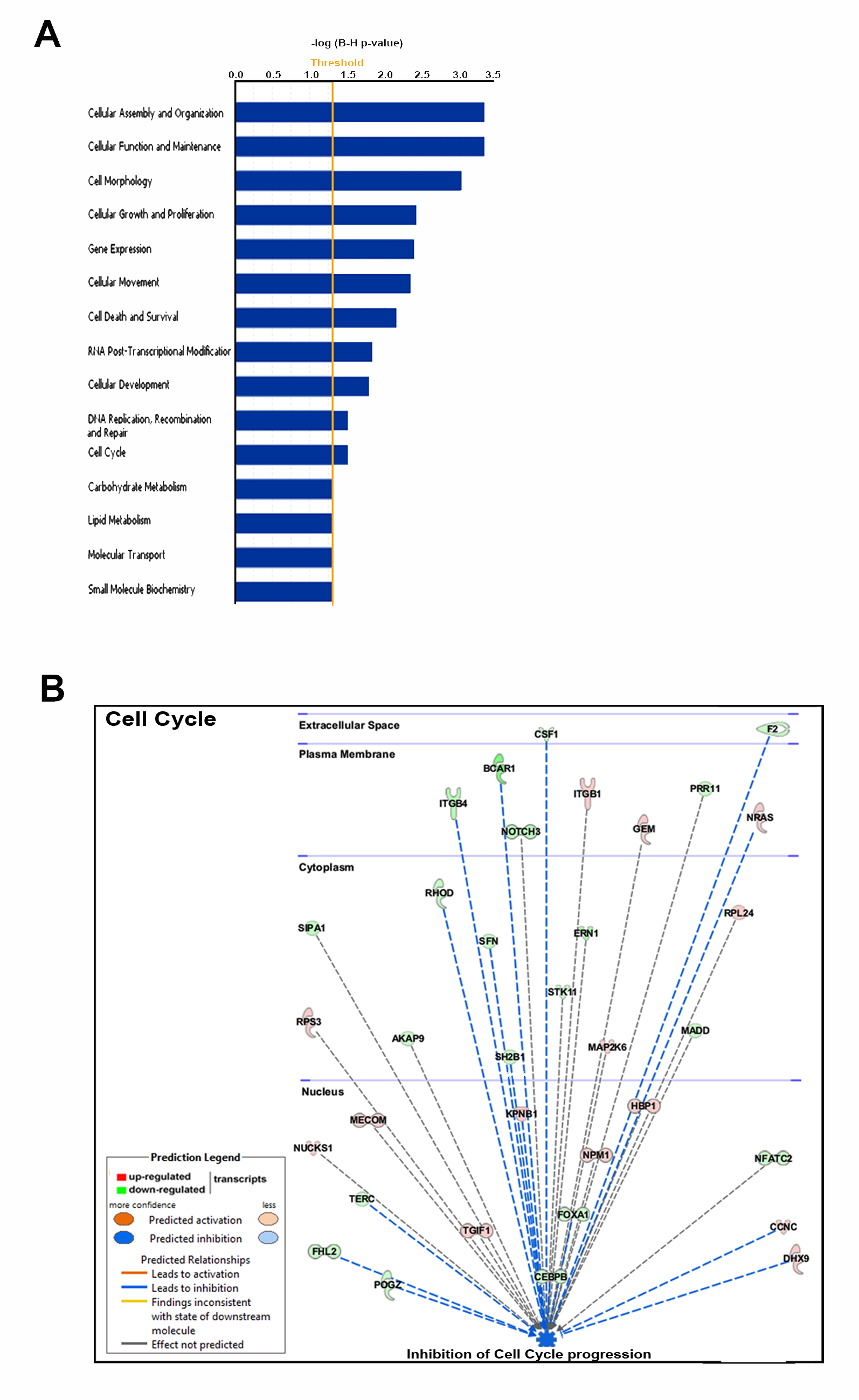
**

**Figure S2**

**
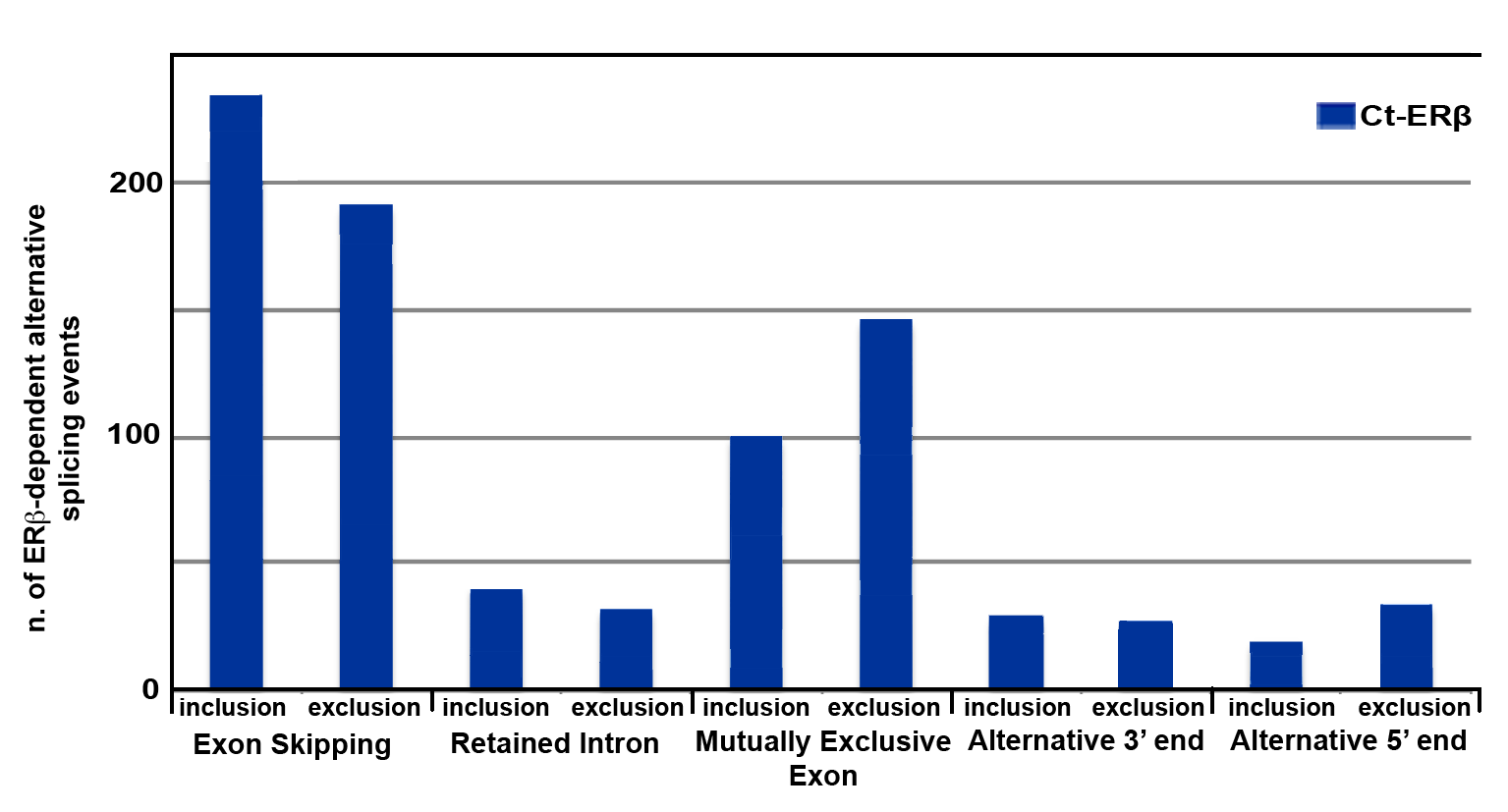
**

**Figure S3**

**
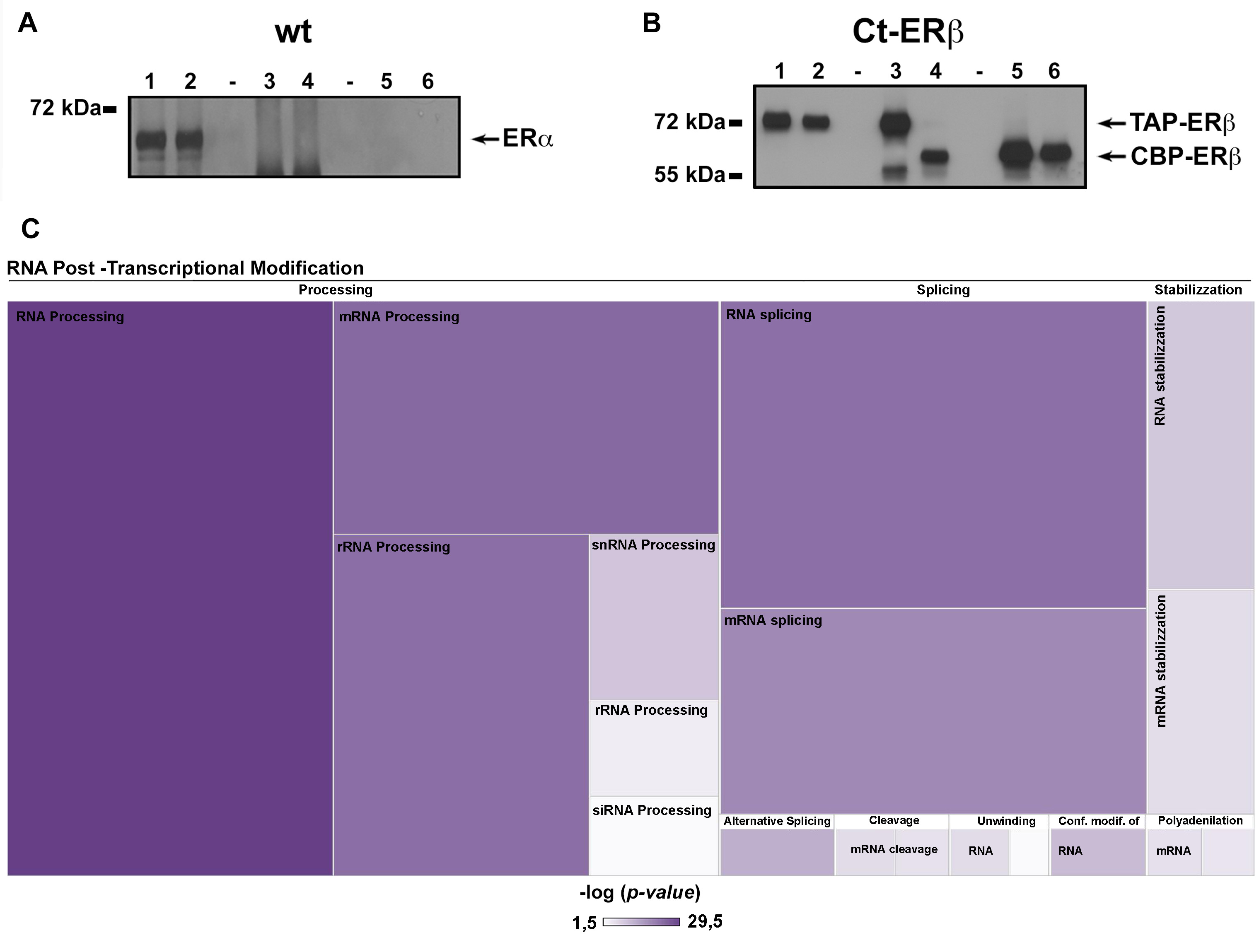
**

**Figure S4**

**
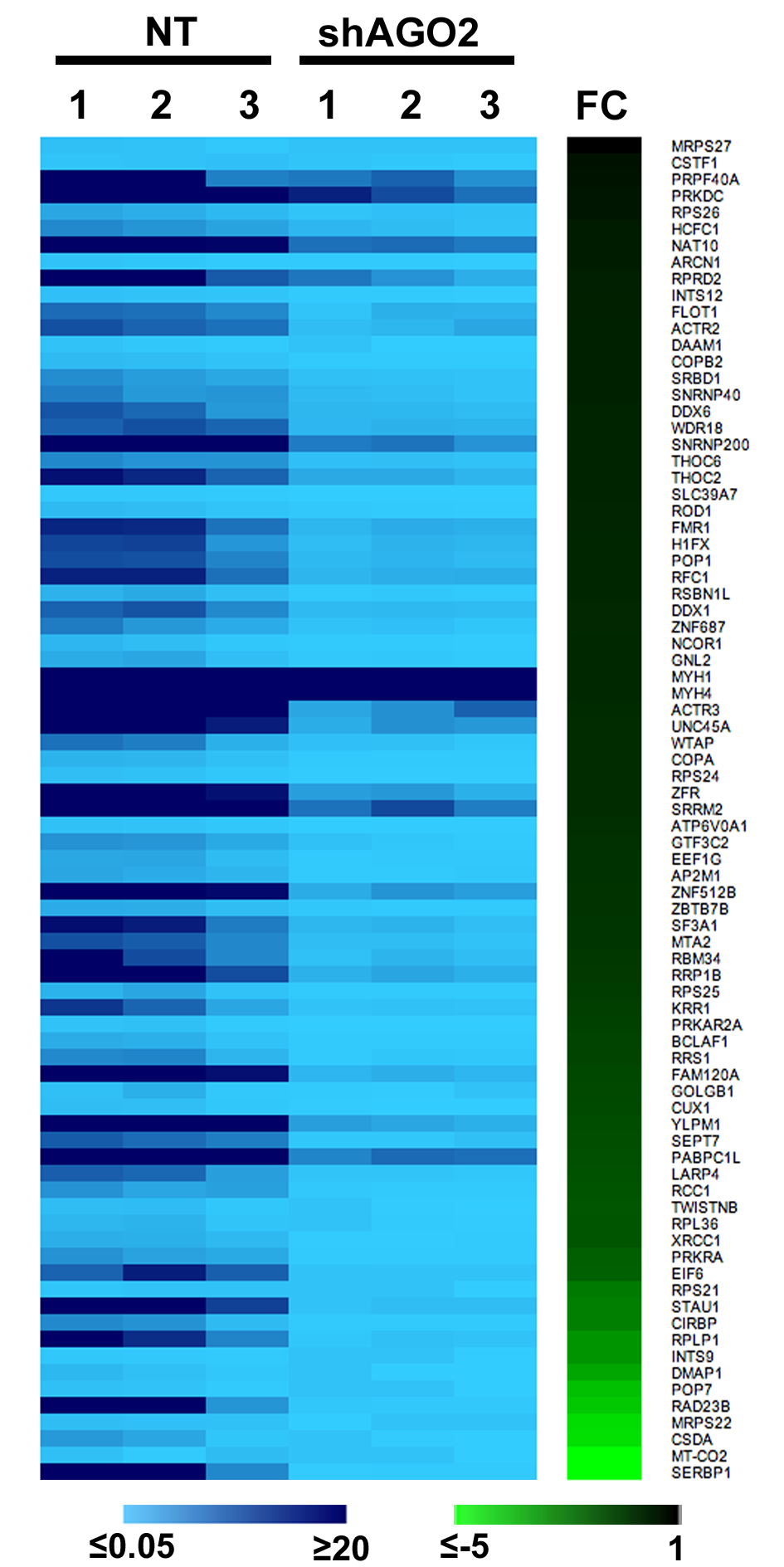
**

**Figure S5**

**
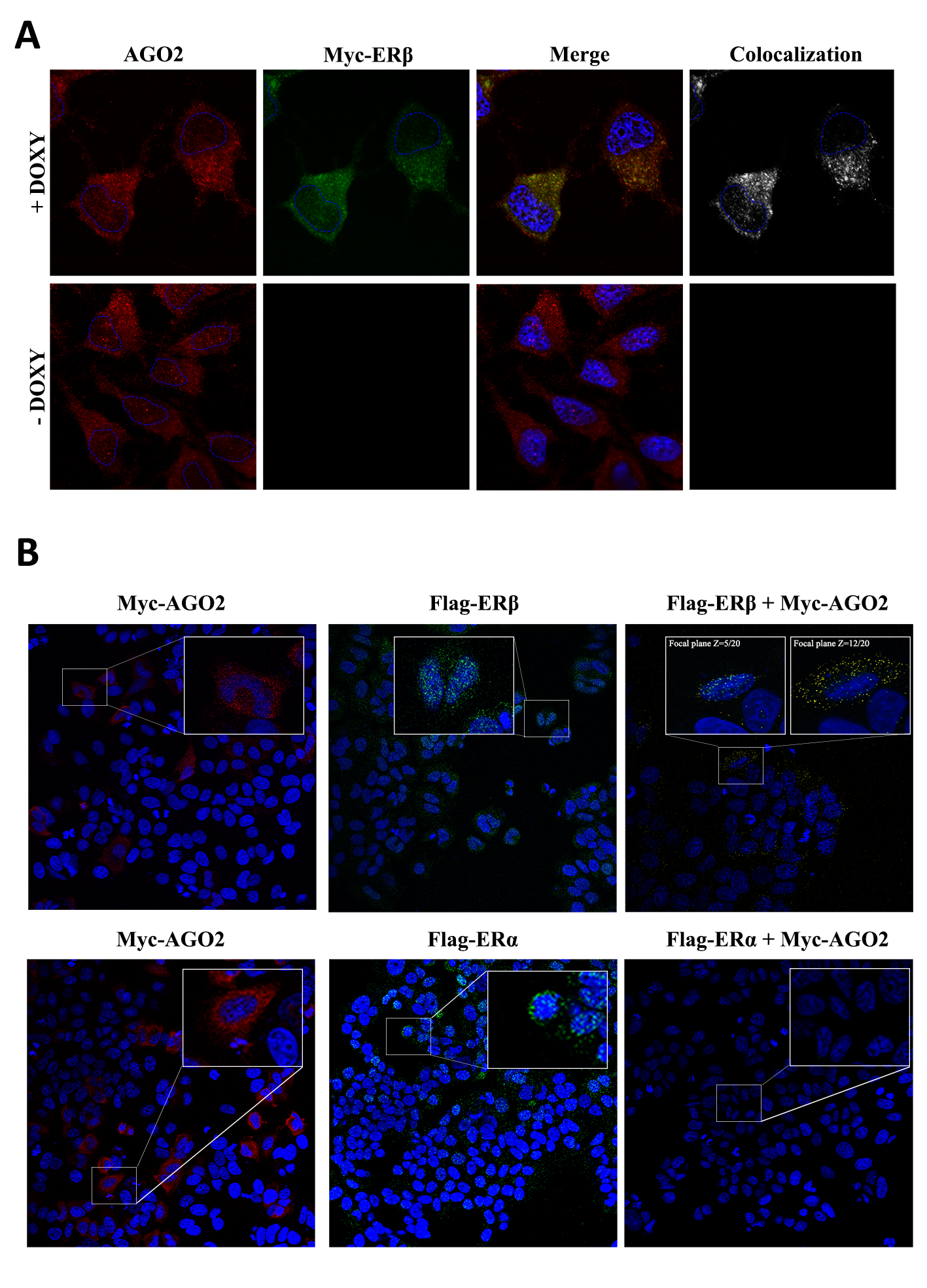
**

**Figure S6**

**
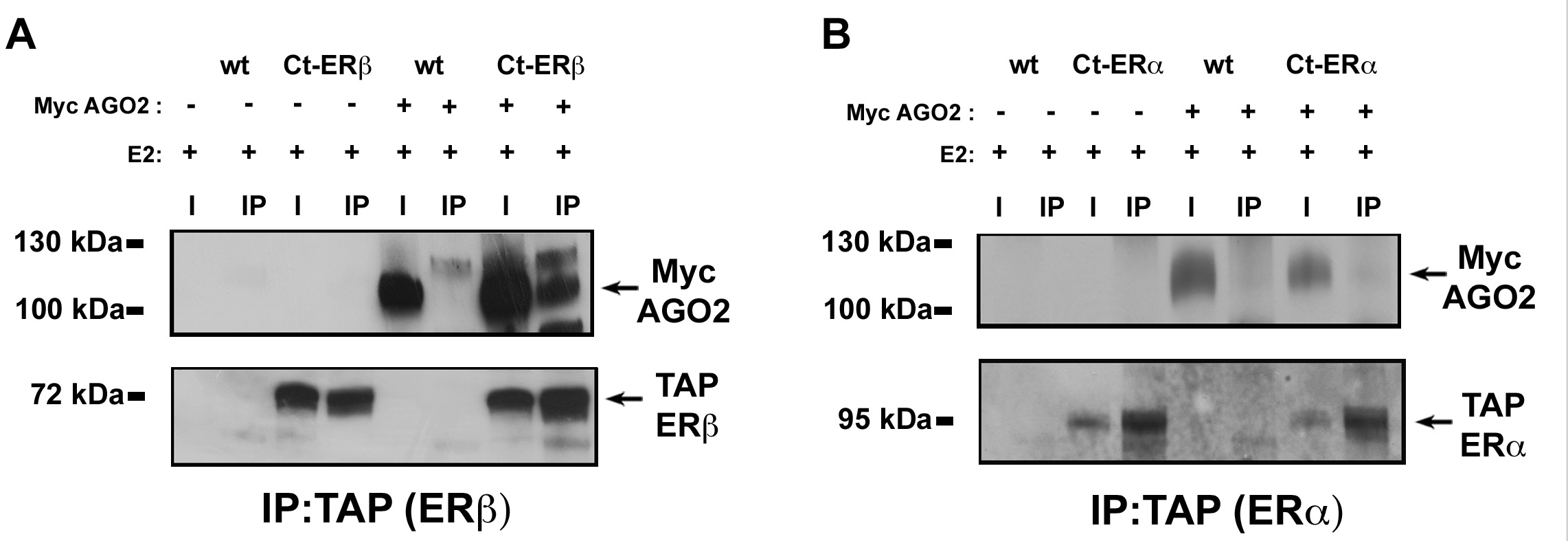
**

**Figure S7**

**
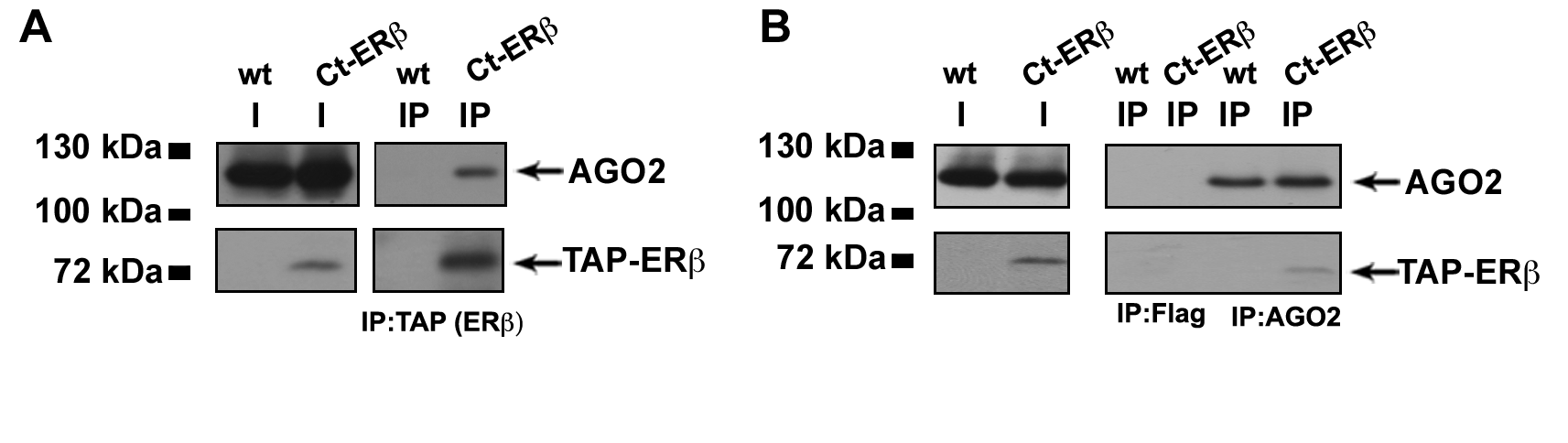
**

**Figure legends**

**Figure S1.** GO analysis of ERβ-regulated transcripts. (**A**) Cellular functions identified by Ingenuity Core analysis (p-value ≤0.05) on ERβ-responsive BC cell genes (FC cut-off |1.5| and FDR≤0.05) harboring an ERβ binding site within the promoter region. (**B**) Detailed representation of the Cell Cycle Interaction network, where red and green symbols mark up- and down-regulated transcripts, respectively, and dotted blu lines indicate the predicted effects on cell cycle progression. Dotted grey line indicate that no effect could be predicted.

**Figure S2.** Bar plot showing alternative splicing events occurring in ERβ-modulated transcripts encoded by loci carrying one or more receptor binding site(s) within the promoter and/or transcription unit in ERβ+ cells (Ct-ERβ). Inclusion and exclusion behavior for each event are shown (FDR ≤0.05; inclusion/exclusion cut-off |0.1|).

**Figure S3.** Representative Western Blot of the different steps of the Tandem Affinity Purification protocol in ERβ-, control cells (wt, panel **A**) and ERβ+ cells (Ct-ERβ, panel **B** ). Lanes 1 and 2: Crude nuclear extracts before and after IgG-Sepharose binding, respectively. Lanes 3 and 4: IgG-Sepharose-bound receptor before and after TEV treatment. Lanes 5 (1st TEV elution) and 6 (2nd TEV elution): samples eluted from IgG-Sepharose. (**C**) Heatmap showing details of the RNA Post-transcriptional Modification function from GO analysis relative to the ERβ-interacting proteins identified here and shown in Figure 3B of the main text.

**Figure S4**. Heatmap showing the amount (expressed as LFQ value normalized respect to ERβ LFQ value) of proteins co-immunoprecipitated with ERβ in control (NT) and AGO2 ‘knock-down’ (shAGO2) cells measured in three biological replicates. FC: average fold-change in shAGO2 *vs* control samples (NT). Only statistically not significant changes in protein content are reported.

**Figure S5.** *In vivo* association of ERβ and AGO2 in BC MCF-7 cells. **A.** ERβ and Ago2 co-localization was evaluated in a cell clone expressing Myc-Flag-tagged ERβ under control of a tet-inducible promoter. The expression patterns of endogenous Argonaute 2 (AGO2) and exogenous ERβ (Myc-ERβ) in the same cell were revealed with specific Abs. Co-localization of the two proteins is revealed by overimposition of the two immunofluorescence signals (Merge) and analysis with ImageJ tool (Colocalization). The test was performed with (+DOXY) and, as negative control, without (-DOXY) tet induction of ERβ expression. **B.** ERβ/Ago2 interaction was evaluated in by proximity ligation assay (PLA). *Upper panels*: expression pattern of myc-tagged Ago2 (left) or flag-tagged ERβ (center) and (right) *in vivo* association between the two proteins in both the nucleus (focal plane Z=5/20) and the extranuclear cell compartment (focal plane Z=12/20). *Lower panels*: expression pattern of myc-tagged Ago2 (left) or flag-tagged ERα (center) and (right) lack of *in vivo* association between the two proteins. Nuclei were stained with DAPI.

**Figure S6.** Effect of estrogen on AGO2 interaction with ERβ or ERα in breast cancer cells. (**A**) Co-immunoprecipitation of ERβ and Ago2 in Ct-ERβ cells transiently transfected with a Myc-Ago2 expression vector in presence of 17beta-estradiol. Not transfected *wt* and Ct-ERβ MCF7 cells were treated in parallel as controls. (**B**) Lack of co-immunoprecipitation of ERβ and Ago2 in Ct-ERα cells transiently transfected with a Myc-Ago2 expression vector in presence of 17beta-estradiol. Not transfected *wt* and Ct-ERβ MCF7 cells were treated in parallel as controls.

**Figure S7.** ERβ-Ago2 interaction on BC cell chromatin. Western Blot performed before and after ChIP in ERβ- (*wt*) and ERβ+ (Ct-ERβ) cells with antibodies against TAP (**A**), Ago2 or Flag (negative control; **B**). In both cases, the first two lanes to the left refer to input (I) samples.
